# Supplementary material for: The mTOR pathway is necessary for survival of mice with short telomeres
Source: Nat Commun. 2020 Mar 3;11:1168. doi: 10.1038/s41467-020-14962-1 (PMC7054554; doi:10.1038/s41467-020-14962-1)
Supplement: Supplementary file 1 — Supplementary information [file 41467_2020_14962_MOESM1_ESM.pdf]

Supplementary Information

The mTOR pathway is necessary for survival of mice with short telomeres

Ferrara-Romeo et al.,

Supplementary Figure 1

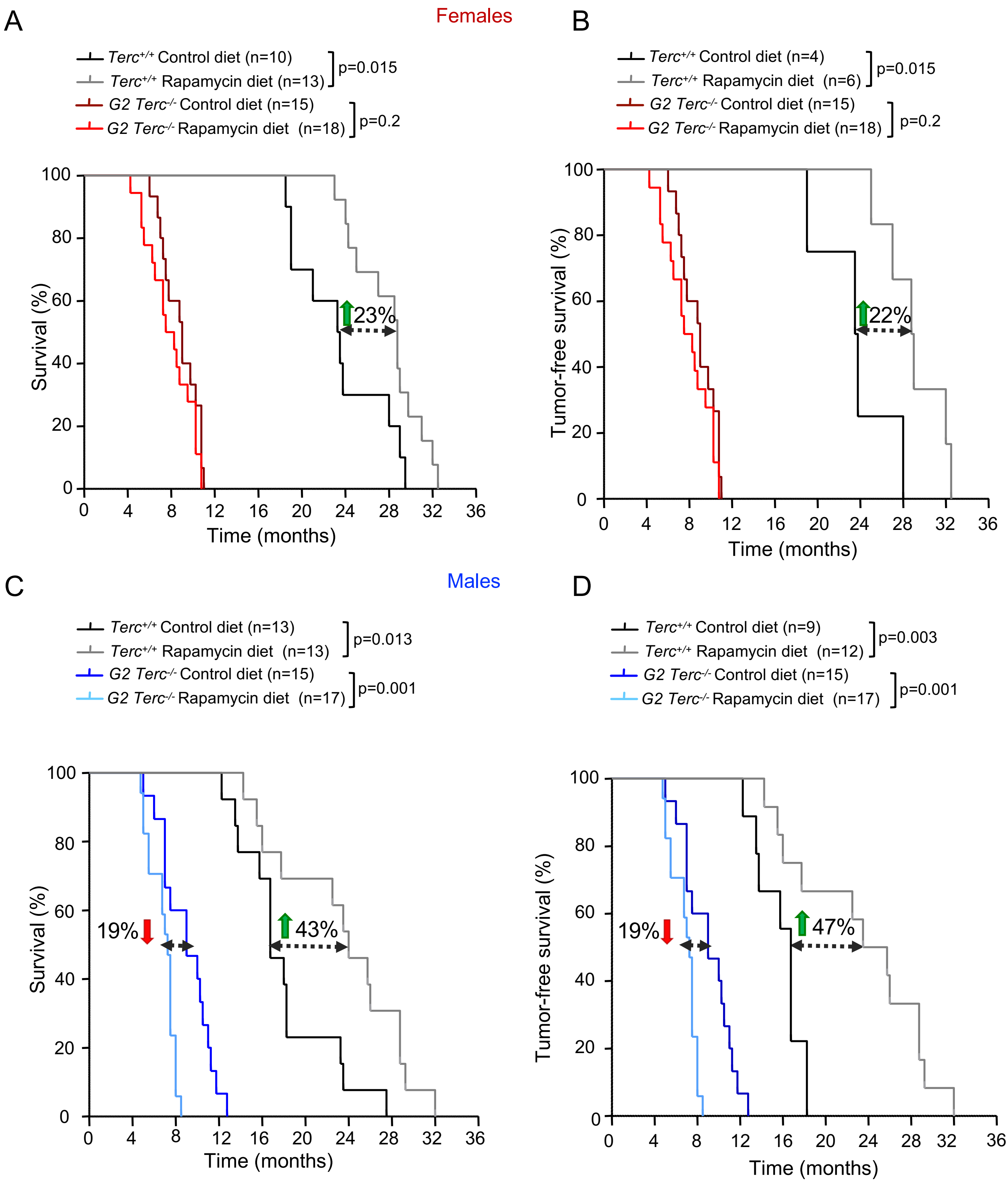

Supplementary Figure 1: Chronic rapamycin treatment shortens the lifespan of G2 *Terc*$^{-/-}$ male mice while does not affect that of female littermates. Kaplan-Meier survival curves of *Terc*$^{+/+}$ and G2 *Terc*$^{-/-}$ female (**A-C**) and male (**B-D**) mice fed rapamycin or control diet. Kaplan-Meier tumor-free survival curves, including only mice that did not present any neoplastic pathology at the time of death are also shown (C,D). The variation of rapamycin fed mice median survival is indicated as percentage of that of the control fed mice of the same genotype; green arrows: rapamycin-mediated increase in median survival; red arrows: rapamycin-mediated decrease in median survival. n= number of mice. Statistical significance was determined by the log rank test. The p-values are indicated. Source data are provided as a Source Data file.

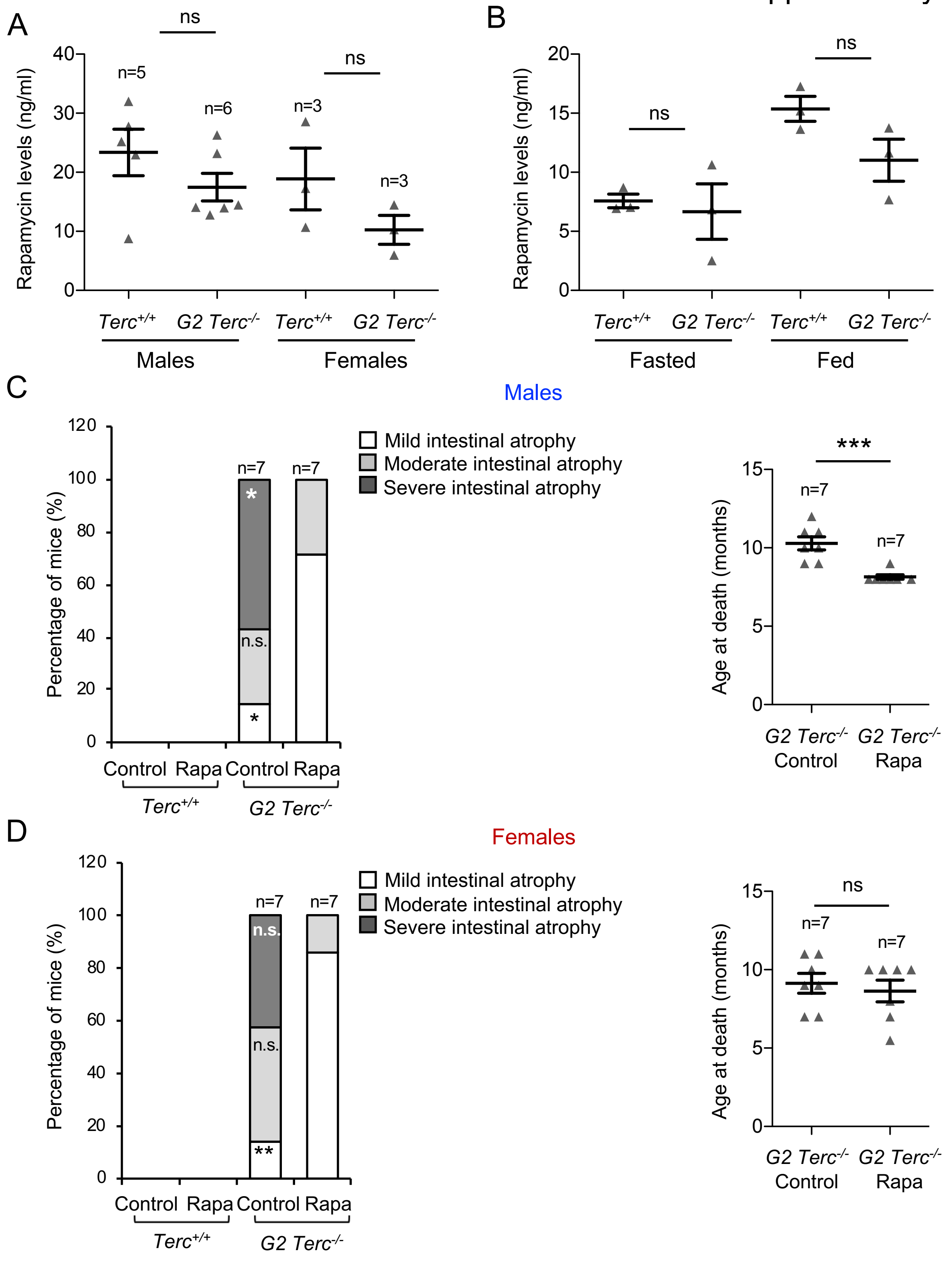

Supplementary Figure 2. Rapamycin treated G2 *Terc*$^{-/-}$ male mice die at an earlier timepoint and show lower severity of intestinal atrophy than the controls. (**A**) Hepatic rapamycin levels in male and female *Terc*$^{+/+}$ and G2 *Terc*$^{-/-}$ mice fed rapamycin diet. (**B**) Plasma rapamycin levels in male *Terc*$^{+/+}$ and G2 *Terc*$^{-/-}$ mice fed rapamycin. Mice were fasted overnight and refed during three hours before sacrifice. Error bars represent the SE. Statistical significance was determined by one-way Anova with post-hoc Tukey test. (**C,D**) Percentage of male (C) and female (D) mice presenting mild, medium or severe intestinal atrophy according to histopathological analysis (left panel). A chi-square test was used to calculate statistical differences in the incidence of intestinal lesions. The age of death of the G2 *Terc*$^{-/-}$ male (C) and female (D) mice fed rapamycin or control diet is shown in the right panels. Error bars represent the SE. A Student's t test was used to calculate the statistical significance. *, $p \leq 0.05$; **, $p \leq 0.01$; ***, $p \leq 0.001$; n.s.= not significant. n= number of mice. Source data are provided as a Source Data file.

Supplementary Figure 3

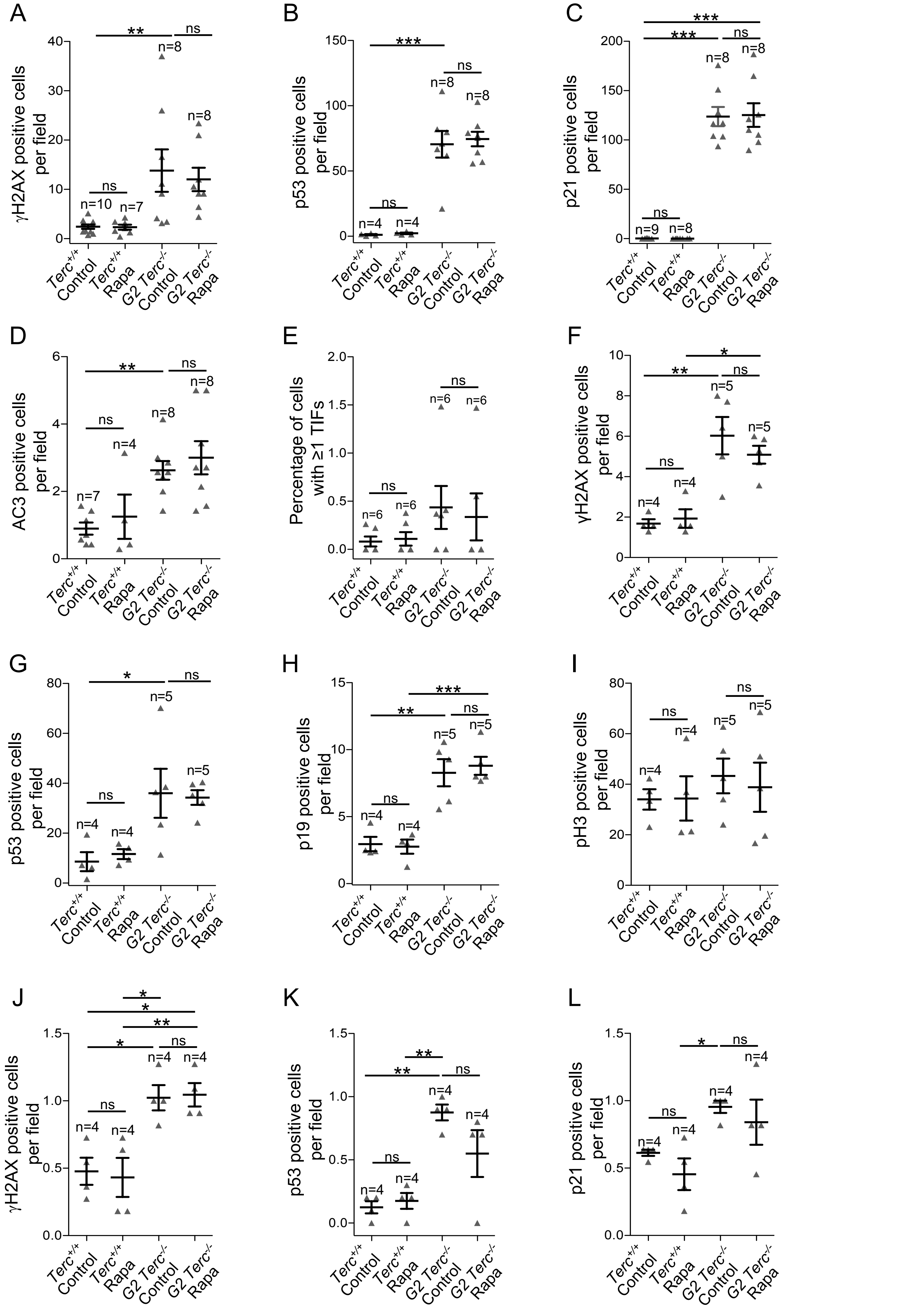

Supplementary Figure 3. Chronic rapamycin treatment does not affect global or telomeric DNA damage in G2 *Terc*$^{-/-}$ mice. (**A-D**) Quantification of γH2AX (A), p53 (B), p21 (C) and AC3 (D) positive cells per field in the crypts of intestine sections from rapamycin or control fed *Terc*$^{+/+}$ and G2 *Terc*$^{-/-}$ mice at the HEP. (**E**) Percentage of cells presenting one or more TRF1 and 53BP1 colocalizing foci (TIFs) in intestine sections from rapamycin or control fed *Terc*$^{+/+}$ and G2 *Terc*$^{-/-}$ mice at the HEP. The age of the wild-type mice analyzed ranges between 20 and 30 months and the age of the G2 *Terc*$^{-/-}$ between 6 and 7 months (A-E). (**F-I**) Quantification of γH2AX (F), p53 (G), p19 (H) and pH3 (I) positive cells per field in the crypts of intestine sections from healthy *Terc*$^{+/+}$ and G2 *Terc*$^{-/-}$ mice fed rapamycin or control diet during 2 months. (**J-L**) Quantification of γH2AX (J), p53 (K) and p21 (L) positive cells per field in skeletal muscle sections from healthy *Terc*$^{+/+}$ and G2 *Terc*$^{-/-}$ mice fed rapamycin or control diet during 2 months. The age of all mice analyzed from both genotypes ranges between 4 and 5 months (F-L). Error bars represent the SE. n= number of mice. Statistical significance was determined by one-way Anova with post-hoc Tukey test. *, $p \leq 0.05$; **, $p \leq 0.01$; ***, $p \leq 0.001$; n.s.= not significant. Source data are provided as a Source Data file.

Supplementary Figure 4

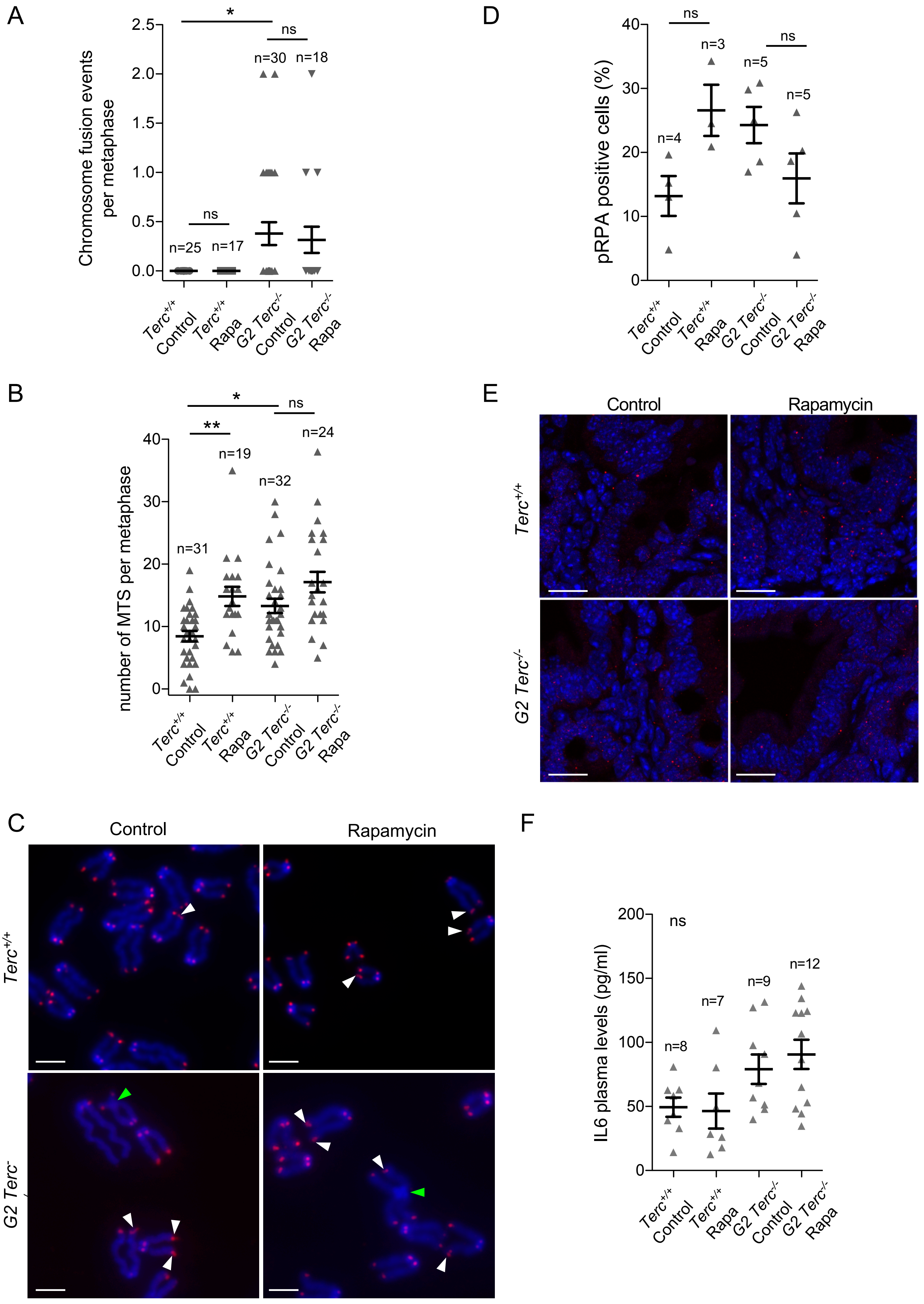

Supplementary Figure 4. Increased levels of replicative damage in rapamycin-treated wild-type mice and MEFs. (**A-C**) Frequency of end to end fusions (A) and multitelomeric signals (MTS) (B) in metaphase spreads from rapamycin treated or untreated *Terc*$^{+/+}$ and G2 *Terc*$^{-/-}$ MEFs. (C) Representative images: white arrowheads indicate MTS; green arrowheads indicate chromosome fusions. Scale bars, 1 μm. n= metaphases used for the analysis from a total of two embryos per condition. (**D-E**) Percentage of cells presenting two or more pRPA foci (D) and representative images (E) in intestine sections from healthy *Terc*$^{+/+}$ and G2 *Terc*$^{-/-}$ mice fed rapamycin or control diet during 2 months. Scale bars, 50 μm. (**F**) Plasma IL-6 levels in rapamycin or control fed *Terc*$^{+/+}$ and G2 *Terc*$^{-/-}$ mice sacrificed at the human end-point. n= number of mice. Error bars represent the SE. Statistical significance was determined by one-way Anova with post-hoc Tukey test. *, $p \leq 0.05$; **, $p \leq 0.01$; n.s.= not significant. Source data are provided as a Source Data file.

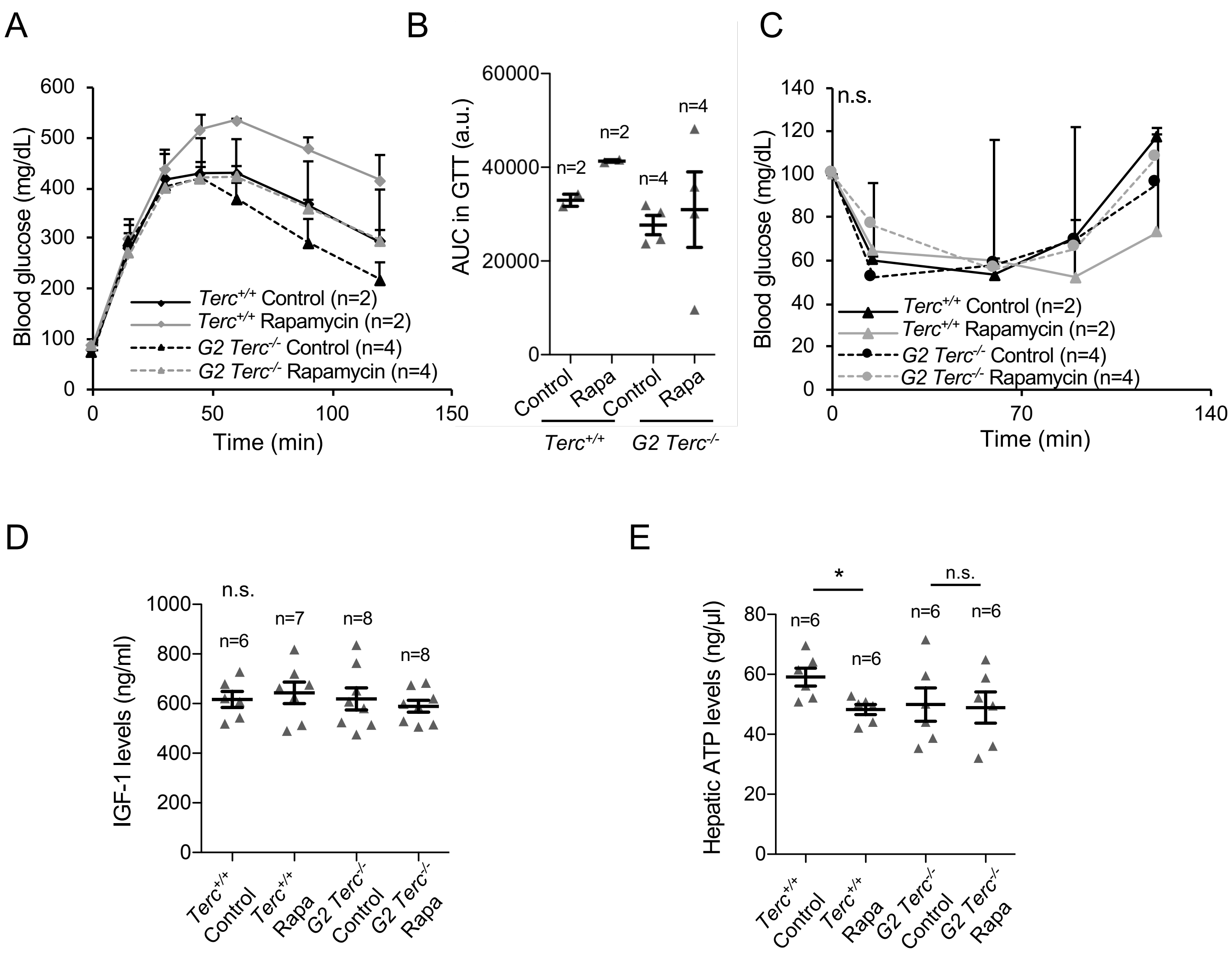

Supplementary Figure 5. Rapamycin treatment does not alter the glucose response in telomerase deficient mice. (**A-C**) Glucose tolerance test (GTT) (A), quantification of the area under the GTT curve (AUC) (B) and Insulin tolerance test (ITT) (C) of *Terc*$^{+/+}$ and G2 *Terc*$^{-/-}$ mice fed with control or rapamycin diet for 5 months. (**D**) IGF1 fasting plasma levels in *Terc*$^{+/+}$ and G2 *Terc*$^{-/-}$ mice fed with control or rapamycin diet for 2 months. (**E**) ATP levels in liver lysates from *Terc*$^{+/+}$ and G2 *Terc*$^{-/-}$ mice fed rapamycin or control diet during 2 months. Error bars represent the SE. n= number of mice. Statistical significance was determined by one-way Anova with post-hoc Tukey test. *, $p \leq 0.05$; **, $p \leq 0.01$; ***, $p \leq 0.001$; n.s.= not significant. Source data are provided as a Source Data file.

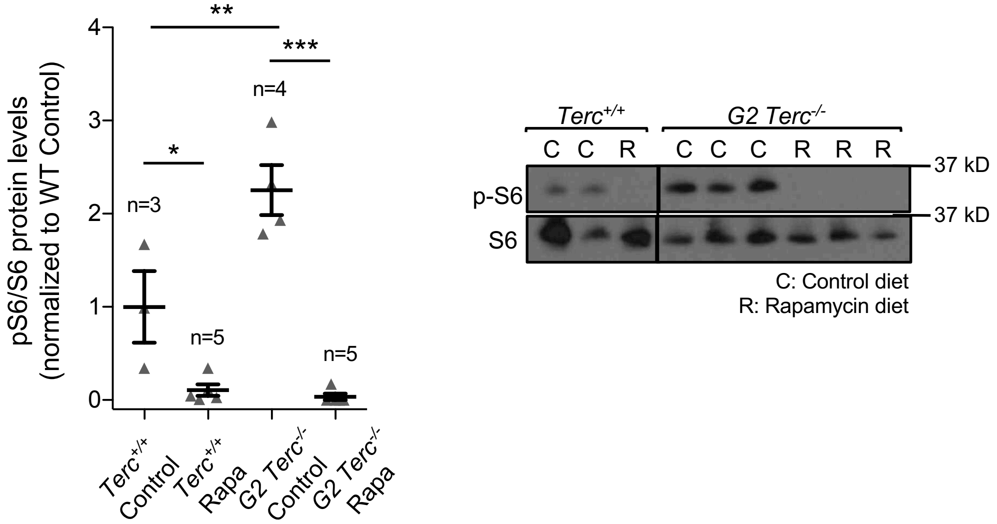

Supplementary Figure 6. Acute rapamycin treatment inhibits hepatic phosphorylation of S6 in both wild-type and telomerase deficient mice. 4 to 6 months old untreated *Terc*$^{+/+}$ and G2 *Terc*$^{-/-}$ male mice were fasted overnight, intraperitoneal injected (i.p.) with vehicle or with rapamycin (2mg/kg body weight) and refed for 1 hour before sacrifice. Western blot quantification (left panel) and representative images (right panel) of pS6 protein levels normalized to total S6 form from hepatic protein extracts. Error bars represent the SE. n= number of mice. Statistical significance was determined by one-way Anova with post-hoc Tukey test. *, $p \leq 0.05$; **, $p \leq 0.01$; ***, $p \leq 0.001$; n.s.= not significant. Source data are provided as a Source Data file.

Supplementary Figure 7

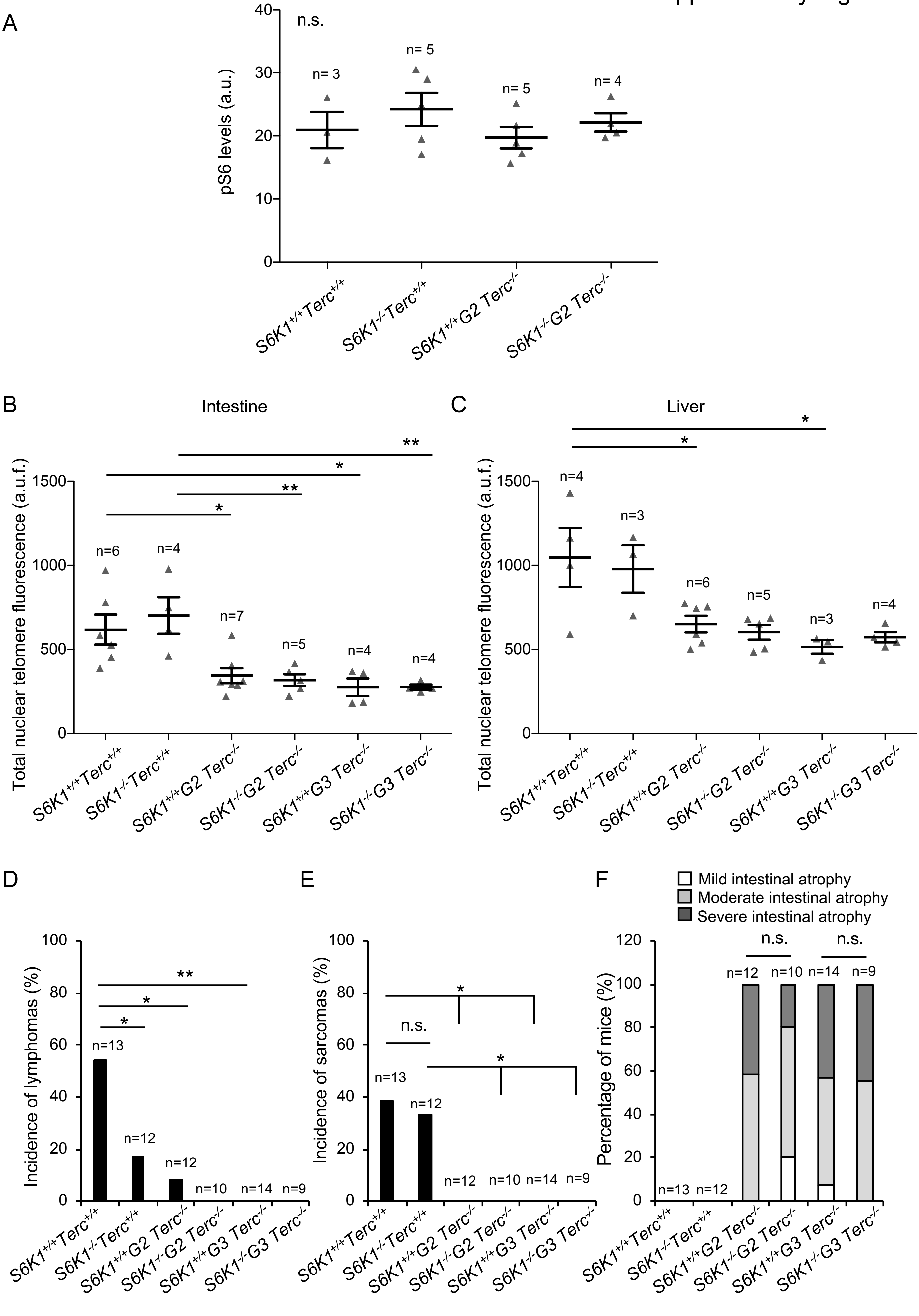

Supplementary Figure 7. Genetic depletion of the mTORC1 target S6K1 has no effects on telomere length. (**A**) Quantification of phosphorylated ribosomal protein S6 (pS6) expression in liver from healthy *S6k1*$^{+/+}$ *Terc*$^{+/+}$, *S6k1*$^{-/-}$ *Terc*$^{+/+}$, *S6k1*$^{+/+}$ G2 *Terc*$^{-/-}$ and *S6k1*$^{-/-}$ G2 *Terc*$^{-/-}$ mice. (**B-C**). Total nuclear telomere fluorescence measured by Q-FISH in intestine (B) and liver (C) sections of *S6k1*$^{+/+}$ *Terc*$^{+/+}$, *S6k1*$^{-/-}$ *Terc*$^{+/+}$, *S6k1*$^{+/+}$ G2 *Terc*$^{-/-}$, *S6k1*$^{-/-}$ G2 *Terc*$^{-/-}$, *S6k1*$^{+/+}$ G3 *Terc*$^{-/-}$ and *S6k1*$^{-/-}$ G3 *Terc*$^{-/-}$ mice at the human endpoint. a.u.f., arbitrary units of fluorescence. Error bars represent the SE. n= number of mice. Statistical significance was determined by one-way Anova with post-hoc Tukey test. (**D-E**) Incidence of lymphomas (D) and sarcomas (E) in *S6k1*$^{+/+}$ *Terc*$^{+/+}$, *S6k1*$^{-/-}$ *Terc*$^{+/+}$, *S6k1*$^{+/+}$ G2 *Terc*$^{-/-}$, *S6k1*$^{-/-}$ G2 *Terc*$^{-/-}$, *S6k1*$^{+/+}$ G3 *Terc*$^{-/-}$ and *S6k1*$^{-/-}$ G3 *Terc*$^{-/-}$ mice at HEP. (**F**) Percentage of mice presenting mild, medium or severe intestinal atrophy according to histopathological analysis. For a detailed histological description see Methods. A chi-square test was used to calculate statistical differences in the incidence of both tumors and intestinal lesions. *, $p \leq 0.05$; **, $p \leq 0.01$; ***, $p \leq 0.001$; n.s.= not significant. Source data are provided as a Source Data file.

Supplementary Figure 8

Figure 4A

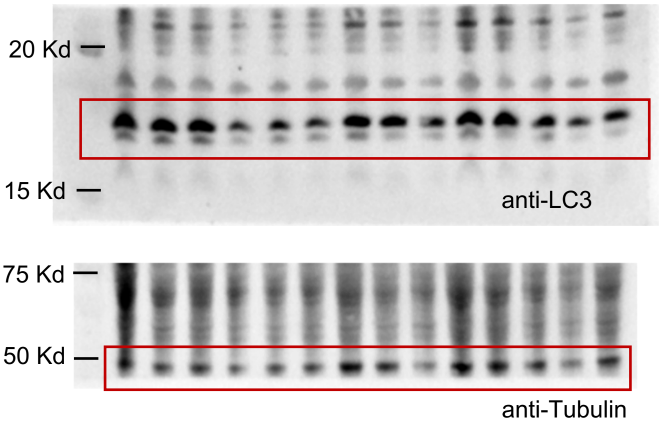

Figure 4D

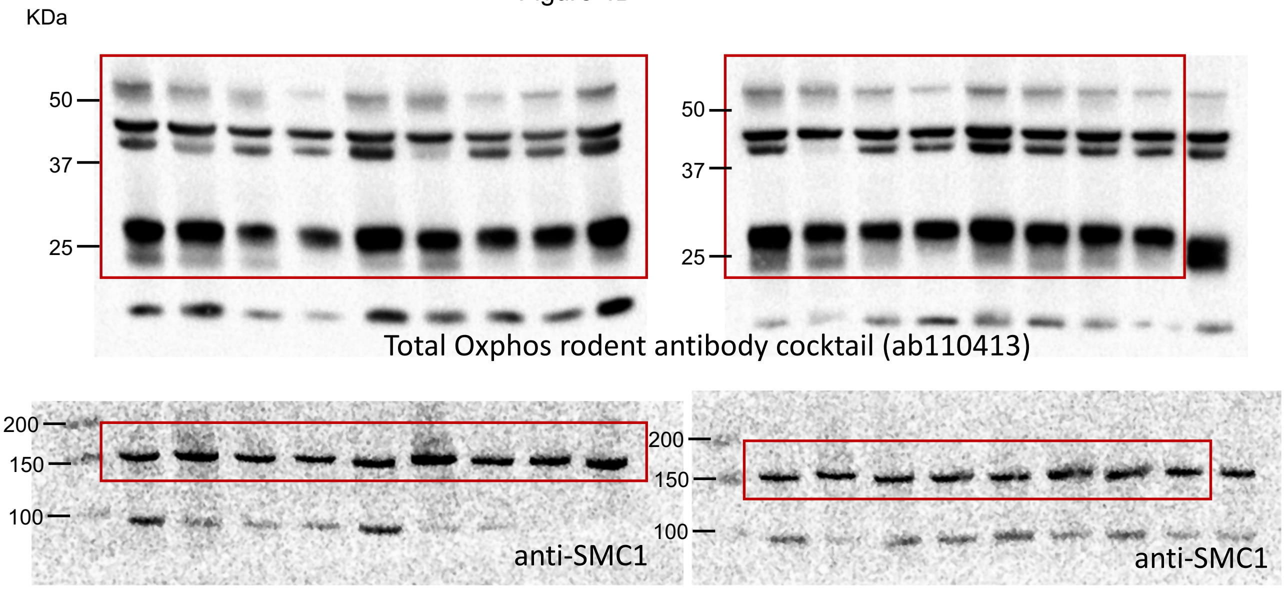

Supplementary Figure 6

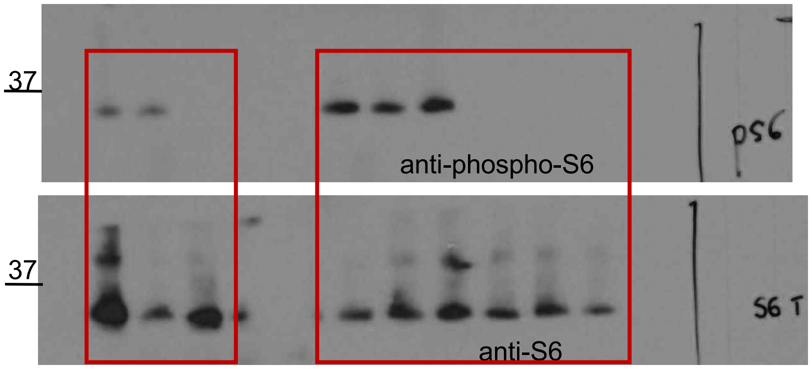

Supplementary Figure 8. Uncropped scans of the western blots presented in main figures as indicated.

Supplementary table 1: Significantly Enriched Gene Sets found in liver of control fed G2*Terc*$^{-/-}$ as compared to control fed *Terc*$^{+/+}$ male mice. Source: KEGG. Related to Figures 5 and 6. FDR: False Discovery Rate, NES: Normalized Enrichment Score. FDR<0.25.

Gene Set Name	Genes	NES	FDR q-val	Control G2 *Terc*$^{-/-}$ status
AMPK SIGNALING PATHWAY	121	3.1648834	0	UP
INSULIN RESISTANCE	106	3.1038256	0	UP
INSULIN SIGNALING PATHWAY	134	3.0586133	0	UP
FATTY ACID METABOLISM	47	2.9452894	0	UP
CARBOHYDRATE DIGESTION AND ABSORPTION	38	2.845864	7.72E-04	UP
PROTEOGLYCANS IN CANCER	189	2.8276849	6.43E-04	UP
TYPE II DIABETES MELLITUS	43	2.7028422	0.00161497	UP
ADIPOCYTOKINE SIGNALING PATHWAY	67	2.5783486	0.00186586	UP
PPAR SIGNALING PATHWAY	76	2.5699723	0.00180003	UP
CHEMOKINE SIGNALING PATHWAY	168	2.5091705	0.00201634	UP
APOPTOSIS	77	2.4757233	0.00249524	UP
BIOSYNTHESIS OF UNSATURATED FATTY ACIDS	24	2.4623764	0.00248634	UP
STEROID BIOSYNTHESIS	19	2.4522338	0.00238349	UP
HEPATITIS C	112	2.359742	0.00565008	UP
FOXO SIGNALING PATHWAY	127	2.3597383	0.00527341	UP
TOLL-LIKE RECEPTOR SIGNALING PATHWAY	85	2.3528357	0.00509434	UP
PEROXISOME	82	2.3483882	0.00509887	UP
NEUROACTIVE LIGAND-RECEPTOR INTERACTION	162	2.3467004	0.00496131	UP
PROPANOATE METABOLISM	30	2.315798	0.00647039	UP
PATHWAYS IN CANCER	365	2.2966464	0.00752849	UP
ALDOSTERONE-REGULATED SODIUM REABSORPTION	32	2.2809405	0.00821323	UP
PYRUVATE METABOLISM	37	2.2509718	0.00918704	UP
FATTY ACID DEGRADATION	48	2.2354846	0.00984842	UP
ALCOHOLISM	138	2.1967294	0.01245044	UP
LEUKOCYTE TRANSENDOTHELIAL MIGRATION	106	2.1933126	0.01215372	UP
ACUTE MYELOID LEUKEMIA	56	2.1715884	0.01329299	UP
CENTRAL CARBON METABOLISM IN CANCER	64	2.1602416	0.01373807	UP

SPHINGOLIPID METABOLISM	45	2.1522489	0.01396554	UP
SPHINGOLIPID SIGNALING PATHWAY	118	2.147515	0.01381939	UP
FATTY ACID ELONGATION	23	2.13176	0.01496293	UP
PROGESTERONE-MEDIATED OOCYTE MATURATION	80	2.0869055	0.01834446	UP
PI3K-AKT SIGNALING PATHWAY	297	2.062528	0.02109587	UP
HEPATITIS B	127	2.0495603	0.02208583	UP
ALPHA-LINOLENIC ACID METABOLISM	17	2.021971	0.02507419	UP
CAMP SIGNALING PATHWAY	166	2.008018	0.02636273	UP
GLUTAMATERGIC SYNAPSE	96	1.9951447	0.02767377	UP
B CELL RECEPTOR SIGNALING PATHWAY	71	1.9874203	0.02794088	UP
PANTOTHENATE AND COA BIOSYNTHESIS	17	1.9787114	0.02858366	UP
REGULATION OF LIPOLYSIS IN ADIPOCYTES	52	1.9739559	0.02871212	UP
FOCAL ADHESION	194	1.9598165	0.03005116	UP
FC EPSILON RI SIGNALING PATHWAY	59	1.9551872	0.03037454	UP
TIGHT JUNCTION	119	1.9495568	0.03052463	UP
ADHERENS JUNCTION	72	1.9033829	0.03816684	UP
AMOEBIASIS	92	1.8995194	0.03819338	UP
RETROGRADE ENDOCANNABINOID SIGNALING	83	1.8952898	0.03838453	UP
OSTEOCLAST DIFFERENTIATION	118	1.883384	0.04009541	UP
MTOR SIGNALING PATHWAY	59	1.8769014	0.04063314	UP
PANCREATIC CANCER	66	1.8455367	0.04690089	UP
VALINE, LEUCINE AND ISOLEUCINE DEGRADATION	48	1.8432689	0.04667575	UP
OOCYTE MEIOSIS	102	1.8426396	0.04598845	UP
NEUROTROPHIN SIGNALING PATHWAY	118	1.8342457	0.04688497	UP
GLUCAGON SIGNALING PATHWAY	91	1.8319644	0.04697597	UP
CIRCADIAN ENTRAINMENT	86	1.8247861	0.0478291	UP
ETHER LIPID METABOLISM	36	1.8215691	0.04777695	UP
GALACTOSE METABOLISM	28	1.7970567	0.05304477	UP
BUTANOATE METABOLISM	24	1.7956797	0.05229379	UP
BILE SECRETION	67	1.7850825	0.0541771	UP
PANCREATIC SECRETION	79	1.7808882	0.05427368	UP

TERPENOID BACKBONE BIOSYNTHESIS	20	1.7763008	0.05459189	UP
NATURAL KILLER CELL MEDIATED CYTOTOXICITY	95	1.7591375	0.05912035	UP
FATTY ACID BIOSYNTHESIS	12	1.7473159	0.06207097	UP
FC GAMMA R-MEDIATED PHAGOCYTOSIS	84	1.7380612	0.0637577	UP
RENAL CELL CARCINOMA	65	1.7320611	0.06462518	UP
CHOLINERGIC SYNAPSE	98	1.7293754	0.06434397	UP
AGE-RAGE SIGNALING PATHWAY IN DIABETIC COMPLICATIONS	98	1.7257478	0.06468777	UP
GLYCOLYSIS / GLUCONEOGENESIS	60	1.7226799	0.06458292	UP
GNRH SIGNALING PATHWAY	80	1.7023908	0.07028724	UP
LYSOSOME	118	1.6840454	0.07642359	UP
NON-SMALL CELL LUNG CANCER	55	1.6668949	0.08241707	UP
PROTEIN DIGESTION AND ABSORPTION	78	1.6662626	0.08145636	UP
ENDOCYTOSIS	251	1.6569936	0.08388225	UP
REGULATION OF ACTIN CYTOSKELETON	196	1.6493038	0.0859137	UP
TASTE TRANSDUCTION	37	1.6469294	0.08572052	UP
ERBB SIGNALING PATHWAY	86	1.6391608	0.08795765	UP
SALIVARY SECRETION	66	1.6318146	0.08981163	UP
SIGNALING PATHWAYS REGULATING PLURIPOTENCY OF STEM CELLS	121	1.6198949	0.0940344	UP
GLYCEROLIPID METABOLISM	51	1.6198658	0.09281316	UP
VEGF SIGNALING PATHWAY	57	1.6159693	0.09305447	UP
DOPAMINERGIC SYNAPSE	112	1.6091005	0.09460286	UP
AMPHETAMINE ADDICTION	55	1.606946	0.09440713	UP
RAS SIGNALING PATHWAY	199	1.5990518	0.09683494	UP
RAP1 SIGNALING PATHWAY	193	1.5898955	0.09995514	UP
ECM-RECEPTOR INTERACTION	77	1.583848	0.10152555	UP
ADRENERGIC SIGNALING IN CARDIOMYOCYTES	131	1.577851	0.10310443	UP
CHAGAS DISEASE (AMERICAN TRYPANOSOMIASIS)	99	1.5519484	0.11530694	UP
MUCIN TYPE O-GLYCAN BIOSYNTHESIS	23	1.5304849	0.12568805	UP
CHRONIC MYELOID LEUKEMIA	73	1.5227218	0.12863262	UP
LONG-TERM DEPRESSION	52	1.5018191	0.1398259	UP
OTHER GLYCAN DEGRADATION	17	1.5007558	0.13879885	UP

CARBON METABOLISM	108	1.4965166	0.13985221	UP
LONG-TERM POTENTIATION	58	1.4819309	0.14662838	UP
GASTRIC ACID SECRETION	62	1.4783543	0.14719985	UP
PROXIMAL TUBULE BICARBONATE RECLAMATION	20	1.4706857	0.15036581	UP
TGF-BETA SIGNALING PATHWAY	80	1.4672668	0.15099029	UP
BACTERIAL INVASION OF EPITHELIAL CELLS	75	1.4585483	0.15479428	UP
LONGEVITY REGULATING PATHWAY MAMMAL	91	1.4355553	0.16908227	UP
GLIOMA	63	1.4259553	0.17415302	UP
GLYCEROPHOSPHOLIPID METABOLISM	83	1.4112378	0.18338785	UP
THYROID HORMONE SIGNALING PATHWAY	112	1.4108173	0.18188746	UP
TNF SIGNALING PATHWAY	105	1.4093287	0.1812987	UP
SMALL CELL LUNG CANCER	82	1.4077882	0.18066047	UP
ENDOCRINE AND OTHER FACTOR-REGULATED CALCIUM REABSORPTION	42	1.4066474	0.17957754	UP
HIF-1 SIGNALING PATHWAY	98	1.381272	0.19779144	UP
PROLACTIN SIGNALING PATHWAY	64	1.3800347	0.19690523	UP
CHOLINE METABOLISM IN CANCER	96	1.3782097	0.19661683	UP
DORSO-VENTRAL AXIS FORMATION	23	1.3738072	0.19817866	UP
NICOTINATE AND NICOTINAMIDE METABOLISM	28	1.3498114	0.21728796	UP
FAT DIGESTION AND ABSORPTION	32	1.3371487	0.22560285	UP
OXYTOCIN SIGNALING PATHWAY	139	1.3360379	0.22441179	UP
VITAMIN DIGESTION AND ABSORPTION	22	1.3360097	0.2223717	UP
TOXOPLASMOSIS	109	1.3343575	0.22191247	UP
REGULATION OF AUTOPHAGY	25	1.3307983	0.22327699	UP
INFLAMMATORY MEDIATOR REGULATION OF TRP CHANNELS	114	1.3062145	0.24394366	UP
ENDOMETRIAL_CANCER	52	1.3043151	0.15899582	UP
SPLICEOSOME	129	-5.164669	0	DOWN
RNA TRANSPORT	152	-4.9536653	0	DOWN
RIBOSOME	127	-4.343727	0	DOWN
RIBOSOME BIOGENESIS IN EUKARYOTES	74	-4.2542067	0	DOWN
PROTEIN PROCESSING IN ENDOPLASMIC RETICULUM	160	-3.280344	0	DOWN
OXIDATIVE PHOSPHORYLATION	110	-2.9752169	0	DOWN

PARKINSON'S DISEASE	119	-2.8635845	1.04E-04	DOWN
ALZHEIMER'S DISEASE	153	-2.5347836	0.00177816	DOWN
HUNTINGTON'S DISEASE	174	-2.4206154	0.00385925	DOWN
PROTEIN EXPORT	25	-2.3177788	0.0065854	DOWN
ANTIGEN PROCESSING AND PRESENTATION	65	-2.3080897	0.00640768	DOWN
HERPES SIMPLEX INFECTION	167	-2.2629187	0.00797792	DOWN
BASAL TRANSCRIPTION FACTORS	40	-2.231337	0.00912519	DOWN
PURINE METABOLISM	160	-2.1688318	0.01324315	DOWN
RETINOL METABOLISM	79	-2.1283448	0.01581899	DOWN
MRNA SURVEILLANCE PATHWAY	83	-2.1159225	0.01604531	DOWN
NON-ALCOHOLIC FATTY LIVER DISEASE (NAFLD)	140	-2.0468614	0.0232909	DOWN
ARGININE BIOSYNTHESIS	19	-2.044629	0.02232879	DOWN
BIOSYNTHESIS OF AMINO ACIDS	71	-1.989957	0.02952518	DOWN
COMPLEMENT AND COAGULATION CASCADES	74	-1.9726043	0.03114636	DOWN
CHEMICAL CARCINOGENESIS	85	-1.9707564	0.03012449	DOWN
AMINOACYL-TRNA BIOSYNTHESIS	43	-1.9594525	0.03052467	DOWN
SYSTEMIC LUPUS ERYTHEMATOSUS	94	-1.9043502	0.04029249	DOWN
P53 SIGNALING PATHWAY	62	-1.7503886	0.09000079	DOWN
TYPE I DIABETES MELLITUS	42	-1.6855992	0.119598	DOWN
CARDIAC MUSCLE CONTRACTION	61	-1.6839485	0.11588509	DOWN
AUTOIMMUNE THYROID DISEASE	38	-1.6812453	0.11319782	DOWN
EPSTEIN-BARR VIRUS INFECTION	188	-1.6721216	0.1142409	DOWN
ASCORBATE AND ALDARATE METABOLISM	26	-1.6499381	0.12298372	DOWN
ALANINE, ASPARTATE AND GLUTAMATE METABOLISM	34	-1.6499135	0.11888426	DOWN
SULFUR RELAY SYSTEM	10	-1.6008209	0.14559743	DOWN
PHENYLALANINE METABOLISM	19	-1.5912888	0.14782467	DOWN
GRAFT-VERSUS-HOST DISEASE	40	-1.5754057	0.15460666	DOWN
PORPHYRIN AND CHLOROPHYLL METABOLISM	40	-1.5421573	0.17505154	DOWN
HYPERTROPHIC CARDIOMYOPATHY (HCM)	72	-1.5392798	0.17238885	DOWN
DRUG METABOLISM OTHER ENZYMES	49	-1.5382695	0.1683792	DOWN
RNA POLYMERASE	29	-1.5271022	0.17293489	DOWN

PRIMARY IMMUNODEFICIENCY	34	-1.5143156	0.17846435	DOWN
TRYPTOPHAN METABOLISM	38	-1.5115862	0.17582451	DOWN
HTLV-I INFECTION	245	-1.5102788	0.1722224	DOWN
STEROID HORMONE BIOSYNTHESIS	73	-1.50436	0.17223918	DOWN
INFLUENZA A	144	-1.4928745	0.17675039	DOWN
ARGININE AND PROLINE METABOLISM	43	-1.4706898	0.19033247	DOWN
HEMATOPOIETIC CELL LINEAGE	74	-1.4426553	0.20906462	DOWN
LYSINE DEGRADATION	50	-1.4402888	0.20665154	DOWN
PROTEASOME	45	-1.4224735	0.21861194	DOWN
ALLOGRAFT REJECTION	37	-1.4112831	0.22410436	DOWN
T CELL RECEPTOR SIGNALING PATHWAY	100	-1.3822106	0.24807143	DOWN
PYRIMIDINE_METABOLISM	97	-1.3747095	0.09202454	DOWN

Supplementary table 2. Significantly Enriched Gene Sets found in liver of rapamycin fed G2*Terc*$^{-/-}$ as compared to rapamycin fed *Terc*$^{+/+}$ male mice. Source: KEGG. Related to Figures 5 and 6. FDR: False Discovery Rate, NES: Normalized Enrichment Score. FDR<0.25.

Gene Set Name	Genes	NES	FDR q-val	Rapamycin G2 *Terc*$^{-/-}$ status
PATHWAYS IN CANCER	356	3.0922444	0	UP
UBIQUITIN MEDIATED PROTEOLYSIS	133	3.0699198	0	UP
PROTEOGLYCANS IN CANCER	188	3.0492885	0	UP
LYSOSOME	118	2.911201	0.00120799	UP
ENDOCYTOSIS	251	2.8641036	9.66E-04	UP
HIPPO SIGNALING PATHWAY	135	2.8210073	8.05E-04	UP
RENAL CELL CARCINOMA	64	2.6611905	0.00187337	UP
INSULIN SIGNALING PATHWAY	131	2.64411	0.00179405	UP
AMPK SIGNALING PATHWAY	120	2.6015248	0.00172369	UP
FOXO SIGNALING PATHWAY	126	2.5149546	0.00283151	UP
COLORECTAL CANCER	61	2.4193528	0.00453569	UP
SPHINGOLIPID SIGNALING PATHWAY	115	2.397904	0.00493088	UP
PHOSPHATIDYLINOSITOL SIGNALING SYSTEM	90	2.389675	0.00482686	UP
INOSITOL PHOSPHATE METABOLISM	67	2.3790843	0.00496363	UP
MICRORNAS IN CANCER	131	2.3545408	0.00550406	UP
MELANOGENESIS	86	2.3483887	0.00546805	UP
FOCAL ADHESION	188	2.3446937	0.00549382	UP
THYROID CANCER	27	2.3193626	0.00630747	UP
AXON GUIDANCE	120	2.3103888	0.00628388	UP
PANCREATIC CANCER	65	2.2796519	0.00730226	UP
ADHERENS JUNCTION	71	2.2611082	0.00763489	UP
ERBB SIGNALING PATHWAY	84	2.256224	0.00760508	UP
PROTEIN PROCESSING IN ENDOPLASMIC RETICULUM	157	2.2309525	0.00832667	UP
ENDOMETRIAL CANCER	51	2.1950536	0.01066368	UP
NEUROTROPHIN SIGNALING PATHWAY	115	2.128319	0.01645012	UP
REGULATION OF ACTIN CYTOSKELETON	193	2.1011975	0.01900827	UP
PI3K-AKT SIGNALING PATHWAY	292	2.0701606	0.02141821	UP

MTOR SIGNALING PATHWAY	59	2.01928	0.02806909	UP
INSULIN RESISTANCE	105	2.0133815	0.02845798	UP
CHRONIC MYELOID LEUKEMIA	73	1.9966896	0.03066756	UP
RAP1 SIGNALING PATHWAY	192	1.9947286	0.02993331	UP
GLIOMA	62	1.9942461	0.02907712	UP
AMINOACYL-TRNA BIOSYNTHESIS	43	1.9834306	0.02972396	UP
GLUCAGON SIGNALING PATHWAY	90	1.9820757	0.02916615	UP
GLYCOSAMINOGLYCAN DEGRADATION	16	1.9643145	0.03191485	UP
TAURINE AND HYPOTAURINE METABOLISM	10	1.9544506	0.03228324	UP
GAP JUNCTION	77	1.9316057	0.03553876	UP
PROSTATE CANCER	85	1.8981462	0.04241204	UP
LONGEVITY REGULATING PATHWAY MAMMAL	92	1.8835485	0.04451259	UP
BACTERIAL INVASION OF EPITHELIAL CELLS	74	1.8804693	0.04423664	UP
SELENOCOMPOUND METABOLISM	17	1.8617716	0.04734533	UP
TGF-BETA SIGNALING PATHWAY	77	1.8443682	0.05002408	UP
GLYCINE, SERINE AND THREONINE METABOLISM	38	1.8370367	0.05064559	UP
CGMP-PKG SIGNALING PATHWAY	151	1.8267882	0.05185489	UP
TIGHT JUNCTION	118	1.8267572	0.05072929	UP
RAS SIGNALING PATHWAY	197	1.8144448	0.05363407	UP
ESTROGEN SIGNALING PATHWAY	90	1.8093607	0.05389243	UP
LONG-TERM POTENTIATION	56	1.8071692	0.05347746	UP
THYROID HORMONE SIGNALING PATHWAY	112	1.8024335	0.05401086	UP
HEDGEHOG SIGNALING PATHWAY	38	1.757571	0.06650731	UP
ALCOHOLISM	136	1.7571137	0.06532154	UP
DOPAMINERGIC SYNAPSE	111	1.7500173	0.06621177	UP
ACUTE MYELOID LEUKEMIA	56	1.7474419	0.06593081	UP
CENTRAL CARBON METABOLISM IN CANCER	62	1.6844167	0.08860525	UP
VEGF SIGNALING PATHWAY	57	1.6710198	0.09312069	UP
OLFACTORY TRANSDUCTION	57	1.6546853	0.09991189	UP
SIGNALING PATHWAYS REGULATING PLURIPOTENCY OF STEM CELLS	116	1.6292119	0.11188322	UP
PHOSPHOLIPASE D SIGNALING PATHWAY	129	1.6267005	0.11106145	UP

AMPHETAMINE ADDICTION	55	1.6186199	0.11337658	UP
SYNAPTIC VESICLE CYCLE	49	1.6175876	0.11228353	UP
MINERAL ABSORPTION	40	1.5926623	0.12492824	UP
REGULATION OF AUTOPHAGY	25	1.5826062	0.12869792	UP
PROPANOATE METABOLISM	30	1.5731058	0.13252166	UP
NON-SMALL CELL LUNG CANCER	56	1.5584964	0.13987306	UP
BIOSYNTHESIS OF AMINO ACIDS	72	1.5485989	0.1441033	UP
ALANINE, ASPARTATE AND GLUTAMATE METABOLISM	32	1.5421727	0.14618537	UP
PROLACTIN SIGNALING PATHWAY	63	1.5364796	0.14793092	UP
WNT SIGNALING PATHWAY	127	1.4900265	0.17983688	UP
PROXIMAL TUBULE BICARBONATE RECLAMATION	19	1.4892727	0.17774917	UP
OOCYTE MEIOSIS	102	1.4798275	0.18206975	UP
GNRH SIGNALING PATHWAY	80	1.4791782	0.18009254	UP
ARGININE BIOSYNTHESIS	19	1.4723141	0.18239404	UP
SMALL CELL LUNG CANCER	81	1.471073	0.1805802	UP
MELANOMA	61	1.4641701	0.18331598	UP
PANCREATIC SECRETION	80	1.4576232	0.18608876	UP
B CELL RECEPTOR SIGNALING PATHWAY	71	1.4503622	0.18947123	UP
N-GLYCAN BIOSYNTHESIS	45	1.4444437	0.19146033	UP
STEROID BIOSYNTHESIS	18	1.4174294	0.21208803	UP
BASAL CELL CARCINOMA	46	1.4034464	0.22240247	UP
ABC TRANSPORTERS	42	1.3962572	0.2261247	UP
HEPATITIS C	111	1.3923653	0.22721793	UP
RENIN SECRETION	56	1.3888859	0.22744657	UP
RIBOSOME	127	-4.7724586	0	DOWN
SPLICEOSOME	129	-3.0106866	3.83E-04	DOWN
AUTOIMMUNE THYROID DISEASE	37	-3.001545	2.56E-04	DOWN
RIBOSOME BIOGENESIS IN EUKARYOTES	73	-2.910773	1.92E-04	DOWN
ALLOGRAFT REJECTION	37	-2.654431	0.00142944	DOWN
OXIDATIVE PHOSPHORYLATION	110	-2.555885	0.00213476	DOWN
HERPES SIMPLEX INFECTION	167	-2.491335	0.00263846	DOWN

VIRAL MYOCARDITIS	57	-2.4787173	0.00271656	DOWN
PARKINSON'S DISEASE	120	-2.467908	0.00251454	DOWN
CARDIAC MUSCLE CONTRACTION	62	-2.4565976	0.00234408	DOWN
STAPHYLOCOCCUS AUREUS INFECTION	47	-2.4169583	0.00279437	DOWN
ALZHEIMER'S DISEASE	152	-2.3433614	0.00464305	DOWN
TYPE I DIABETES MELLITUS	44	-2.2602105	0.0069969	DOWN
GRAFT-VERSUS-HOST DISEASE	41	-2.2524831	0.00678538	DOWN
SYSTEMIC LUPUS ERYTHEMATOSUS	94	-2.242727	0.00708538	DOWN
PRIMARY IMMUNODEFICIENCY	32	-2.2163231	0.00824552	DOWN
HUNTINGTON'S DISEASE	170	-2.1747596	0.01079827	DOWN
NON-ALCOHOLIC FATTY LIVER DISEASE (NAFLD)	139	-2.1712675	0.01028474	DOWN
ANTIGEN PROCESSING AND PRESENTATION	65	-2.1423728	0.01180709	DOWN
ARACHIDONIC ACID METABOLISM	72	-2.072558	0.01817685	DOWN
DNA REPLICATION	35	-2.0440545	0.0206175	DOWN
INTESTINAL IMMUNE NETWORK FOR IGA PRODUCTION	35	-2.038283	0.02043811	DOWN
CHEMICAL CARCINOGENESIS	79	-1.8775358	0.05123869	DOWN
BASE EXCISION REPAIR	33	-1.8669465	0.0523097	DOWN
GLYCOSYLPHOSPHATIDYLINOSITOL(GPI)-ANCHOR BIOSYNTHESIS	25	-1.8513412	0.05515993	DOWN
PHAGOSOME	141	-1.8377446	0.05725375	DOWN
JAK-STAT SIGNALING PATHWAY	123	-1.8318197	0.05720863	DOWN
ASTHMA	14	-1.82212	0.05793134	DOWN
DRUG METABOLISM CYTOCHROME P450	57	-1.8087647	0.05933829	DOWN
RETINOL METABOLISM	79	-1.7806334	0.06793026	DOWN
BLADDER CANCER	38	-1.7781636	0.0663915	DOWN
HEMATOPOIETIC CELL LINEAGE	75	-1.7458186	0.07699523	DOWN
NEUROACTIVE LIGAND-RECEPTOR INTERACTION	154	-1.7129807	0.08858093	DOWN
NICOTINE ADDICTION	23	-1.6902275	0.09682868	DOWN
BASAL TRANSCRIPTION FACTORS	40	-1.6823457	0.09769311	DOWN
TOLL-LIKE RECEPTOR SIGNALING PATHWAY	86	-1.652589	0.10985594	DOWN
METABOLISM OF XENOBIOTICS BY CYTOCHROME P450	59	-1.6497886	0.10820282	DOWN
RNA TRANSPORT	151	-1.6048307	0.13143615	DOWN

LEISHMANIASIS	61	-1.5986427	0.13163061	DOWN
CYTOKINE-CYTOKINE RECEPTOR INTERACTION	196	-1.5751444	0.14314534	DOWN
FATTY ACID DEGRADATION	48	-1.5071745	0.19141929	DOWN
OSTEOCLAST DIFFERENTIATION	115	-1.4879378	0.20262352	DOWN
PPAR SIGNALING PATHWAY	74	-1.4869812	0.19876748	DOWN
MATURITY ONSET DIABETES OF THE YOUNG	15	-1.4805155	0.19928062	DOWN
CELL ADHESION MOLECULES (CAMS)	127	-1.467468	0.20641698	DOWN
INFLUENZA A	148	-1.4564617	0.21099645	DOWN
FATTY ACID ELONGATION	23	-1.447815	0.21460947	DOWN
GLUTATHIONE METABOLISM	51	-1.4441649	0.21377355	DOWN
P53 SIGNALING PATHWAY	62	-1.4173028	0.23632577	DOWN
ALPHA-LINOLENIC ACID METABOLISM	18	-1.4169383	0.23186351	DOWN

Supplementary table 3. Significantly Enriched Gene Sets found in liver of rapamycin fed G2 *Terc*$^{-/-}$ as compared to control fed G2 *Terc*$^{-/-}$ male mice. Source: KEGG. Related to Figure 6. FDR: False Discovery Rate, NES: Normalized Enrichment Score. FDR<0.25.

Gene Set Name	Genes	NES	FDR q-val	Rapamycin G2 *Terc*$^{-/-}$ status
PROTEIN PROCESSING IN ENDOPLASMIC RETICULUM	157	0.28916815	0	UP
SELENOCOMPOUND METABOLISM	17	0.49278027	0.0061158	UP
ARGININE BIOSYNTHESIS	19	0.47077602	0.00520504	UP
AMINOACYL-TRNA BIOSYNTHESIS	43	0.3091777	0.00639301	UP
SPLICEOSOME	129	0.17138207	0.01862543	UP
UBIQUITIN MEDIATED PROTEOLYSIS	134	0.16827448	0.01629518	UP
RNA TRANSPORT	153	0.14787395	0.02323	UP
PROTEIN EXPORT	25	0.3601416	0.02176904	UP
ALANINE, ASPARTATE AND GLUTAMATE METABOLISM	34	0.2771714	0.06492831	UP
FANCONI ANEMIA PATHWAY	48	0.23804267	0.06230341	UP
BIOSYNTHESIS OF AMINO ACIDS	71	0.18671805	0.08951562	UP
PORPHYRIN AND CHLOROPHYLL METABOLISM	40	0.2373626	0.1048801	UP
RIBOSOME BIOGENESIS IN EUKARYOTES	75	0.17106271	0.12850201	UP
RNA DEGRADATION	72	0.17004296	0.14830698	UP
GLUCAGON SIGNALING PATHWAY	91	0.15078638	0.1385388	UP
REGULATION OF AUTOPHAGY	25	0.28338012	0.13444786	UP
CYSTEINE AND METHIONINE METABOLISM	41	0.21572748	0.18022937	UP
RIBOSOME	127	-5.9572296	0	DOWN
BIOSYNTHESIS OF UNSATURATED FATTY ACIDS	24	-3.7971115	0	DOWN
FATTY ACID ELONGATION	22	-3.522607	0	DOWN
CELL ADHESION MOLECULES (CAMS)	129	-3.3829486	0	DOWN
PPAR SIGNALING PATHWAY	75	-3.1450117	0	DOWN
FATTY ACID METABOLISM	47	-3.1243455	0	DOWN
CYTOKINE-CYTOKINE RECEPTOR INTERACTION	200	-3.082192	0	DOWN
NEUROACTIVE LIGAND-RECEPTOR INTERACTION	155	-3.0274885	0	DOWN
INTESTINAL IMMUNE NETWORK FOR IGA PRODUCTION	37	-2.9076881	0	DOWN
ECM-RECEPTOR INTERACTION	75	-2.9028537	1.57E-04	DOWN

B CELL RECEPTOR SIGNALING PATHWAY	71	-2.8316715	1.43E-04	DOWN
ARACHIDONIC ACID METABOLISM	73	-2.8227	2.46E-04	DOWN
PEROXISOME	82	-2.8000884	2.27E-04	DOWN
STAPHYLOCOCCUS AUREUS INFECTION	47	-2.7451718	4.07E-04	DOWN
PI3K-AKT SIGNALING PATHWAY	297	-2.6925855	5.71E-04	DOWN
AMOEBIASIS	94	-2.6733074	6.29E-04	DOWN
FOCAL ADHESION	190	-2.6604712	5.92E-04	DOWN
ALZHEIMER'S DISEASE	153	-2.6294131	7.20E-04	DOWN
GLUTAMATERGIC SYNAPSE	95	-2.5457013	0.00106414	DOWN
PROPANOATE METABOLISM	30	-2.5041447	0.0012135	DOWN
AGE-RAGE SIGNALING PATHWAY IN DIABETIC COMPLICATIONS	98	-2.4691036	0.00151293	DOWN
OXIDATIVE PHOSPHORYLATION	110	-2.465208	0.00144416	DOWN
OSTEOCLAST DIFFERENTIATION	116	-2.4506807	0.00164331	DOWN
CARDIAC MUSCLE CONTRACTION	59	-2.4447184	0.00157484	DOWN
PROTEIN DIGESTION AND ABSORPTION	75	-2.4240127	0.00209343	DOWN
LEISHMANIASIS	63	-2.399506	0.00279851	DOWN
HEMATOPOIETIC CELL LINEAGE	75	-2.34161	0.00433122	DOWN
PYRUVATE METABOLISM	37	-2.332685	0.00433343	DOWN
PARKINSON'S DISEASE	118	-2.2719283	0.00587592	DOWN
INFLAMMATORY BOWEL DISEASE (IBD)	51	-2.2474272	0.00644605	DOWN
MALARIA	40	-2.1998763	0.00928841	DOWN
LEUKOCYTE TRANSENDOTHELIAL MIGRATION	104	-2.1023579	0.01754786	DOWN
SMALL CELL LUNG CANCER	82	-2.0950367	0.017496	DOWN
RAP1 SIGNALING PATHWAY	194	-2.092141	0.01728199	DOWN
VIRAL MYOCARDITIS	57	-2.080474	0.01828792	DOWN
FC GAMMA R-MEDIATED PHAGOCYTOSIS	85	-2.0731738	0.01854522	DOWN
DRUG METABOLISM CYTOCHROME P450	60	-2.0452688	0.02091188	DOWN
FATTY ACID DEGRADATION	48	-2.0367556	0.02157856	DOWN
GLYCOSAMINOGLYCAN DEGRADATION	16	-2.0293064	0.02200365	DOWN
RHEUMATOID ARTHRITIS	71	-2.0265129	0.0216303	DOWN
ADRENERGIC SIGNALING IN CARDIOMYOCYTES	127	-2.0240831	0.02121093	DOWN

NICOTINE ADDICTION	21	-2.0109947	0.02274035	DOWN
TASTE TRANSDUCTION	39	-2.0094464	0.02237786	DOWN
CHAGAS DISEASE (AMERICAN TRYPANOSOMIASIS)	100	-1.9934084	0.02365954	DOWN
APOPTOSIS	77	-1.9728596	0.02629174	DOWN
HUNTINGTON'S DISEASE	171	-1.9713728	0.02615034	DOWN
NON-ALCOHOLIC FATTY LIVER DISEASE (NAFLD)	140	-1.9697707	0.02590848	DOWN
ASTHMA	15	-1.9647033	0.02616034	DOWN
PENTOSE PHOSPHATE PATHWAY	26	-1.9554191	0.02717714	DOWN
CHEMOKINE SIGNALING PATHWAY	167	-1.934532	0.03037396	DOWN
TYPE II DIABETES MELLITUS	41	-1.933179	0.0298943	DOWN
ALPHA-LINOLENIC ACID METABOLISM	17	-1.9247615	0.03067989	DOWN
PHOSPHOLIPASE D SIGNALING PATHWAY	128	-1.9230334	0.03033683	DOWN
GLUTATHIONE METABOLISM	52	-1.8924209	0.0356763	DOWN
RAS SIGNALING PATHWAY	199	-1.8905113	0.035459	DOWN
PLATELET ACTIVATION	116	-1.8888265	0.03520342	DOWN
TYPE I DIABETES MELLITUS	44	-1.8747177	0.03824291	DOWN
CALCIUM SIGNALING PATHWAY	147	-1.8728307	0.03808006	DOWN
CENTRAL CARBON METABOLISM IN CANCER	62	-1.8651986	0.03913321	DOWN
PROTEOGLYCANS IN CANCER	187	-1.8499032	0.04206407	DOWN
CHOLINERGIC SYNAPSE	96	-1.8393053	0.04375595	DOWN
RETROGRADE ENDOCANNABINOID SIGNALING	77	-1.8388911	0.04309833	DOWN
SPHINGOLIPID METABOLISM	45	-1.832876	0.04385966	DOWN
TOXOPLASMOSIS	109	-1.8195944	0.04632829	DOWN
CHEMICAL CARCINOGENESIS	83	-1.8190092	0.04566003	
AUTOIMMUNE THYROID DISEASE	38	-1.8114239	0.04681086	DOWN
GRAFT-VERSUS-HOST DISEASE	42	-1.7931727	0.05026326	DOWN
FC EPSILON RI SIGNALING PATHWAY	57	-1.788697	0.05051804	DOWN
AMPK SIGNALING PATHWAY	121	-1.7844503	0.05087442	DOWN
FAT DIGESTION AND ABSORPTION	32	-1.7825629	0.05070502	DOWN
LYSOSOME	118	-1.7825615	0.04999086	DOWN
AMINO SUGAR AND NUCLEOTIDE SUGAR METABOLISM	47	-1.7769676	0.05089381	DOWN

SALIVARY SECRETION	63	-1.7736106	0.05144415	DOWN
INFLAMMATORY MEDIATOR REGULATION OF TRP CHANNELS	112	-1.7654241	0.05301827	DOWN
ALDOSTERONE-REGULATED SODIUM REABSORPTION	33	-1.7603239	0.05351333	DOWN
REGULATION OF LIPOLYSIS IN ADIPOCYTES	52	-1.7476665	0.05667255	DOWN
BETA-ALANINE METABOLISM	30	-1.7238033	0.06339982	DOWN
CARBOHYDRATE DIGESTION AND ABSORPTION	38	-1.7179928	0.06454758	DOWN
CGMP-PKG SIGNALING PATHWAY	152	-1.7057676	0.06778571	DOWN
CIRCADIAN ENTRAINMENT	84	-1.7054437	0.06705159	DOWN
TGF-BETA SIGNALING PATHWAY	79	-1.7032138	0.06694289	DOWN
TOLL-LIKE RECEPTOR SIGNALING PATHWAY	86	-1.6994646	0.06734918	DOWN
INSULIN RESISTANCE	106	-1.6954939	0.06782302	DOWN
HTLV-I INFECTION	244	-1.6820043	0.07138252	DOWN
ETHER LIPID METABOLISM	36	-1.6814336	0.07076459	DOWN
METABOLISM OF XENOBIOTICS BY CYTOCHROME P450	61	-1.6613115	0.07739313	DOWN
DILATED CARDIOMYOPATHY	75	-1.6440519	0.08345588	DOWN
RETINOL METABOLISM	79	-1.6420301	0.0833463	DOWN
MICRORNAS IN CANCER	133	-1.6212658	0.09149841	DOWN
ALLOGRAFT REJECTION	38	-1.6117754	0.09449264	DOWN
REGULATION OF ACTIN CYTOSKELETON	196	-1.5921072	0.10267066	DOWN
VALINE, LEUCINE AND ISOLEUCINE DEGRADATION	49	-1.5883764	0.1033064	DOWN
LINOLEIC ACID METABOLISM	37	-1.579916	0.10607211	DOWN
PANCREATIC SECRETION	82	-1.556627	0.11674722	DOWN
GLYCOSAMINOGLYCAN BIOSYNTHESIS CHONDROITIN SULFATE / DERMATAN SULFATE	20	-1.5519258	0.1181047	DOWN
PRION DISEASES	34	-1.5384951	0.12439484	DOWN
GLYCOSAMINOGLYCAN BIOSYNTHESIS KERATAN SULFATE	13	-1.5363069	0.12430619	DOWN
GLYCEROLIPID METABOLISM	51	-1.5333852	0.1244705	DOWN
SPHINGOLIPID SIGNALING PATHWAY	116	-1.5328959	0.12342775	DOWN
ARGININE AND PROLINE METABOLISM	43	-1.5284848	0.12433367	DOWN
AMPHETAMINE ADDICTION	54	-1.5132216	0.13125388	DOWN
CHOLINE METABOLISM IN CANCER	95	-1.5027573	0.13661873	DOWN

TUBERCULOSIS	154	-1.5011954	0.13611844	DOWN
DRUG METABOLISM OTHER ENZYMES	49	-1.4853066	0.14478897	DOWN
MAPK SIGNALING PATHWAY	227	-1.4724092	0.15186915	DOWN
TIGHT JUNCTION	115	-1.4681963	0.15362424	DOWN
NATURAL KILLER CELL MEDIATED CYTOTOXICITY	96	-1.4639932	0.15485127	DOWN
GLYCOSPHINGOLIPID BIOSYNTHESIS LACTO AND NEOLACTO SERIES	20	-1.4591753	0.1567413	DOWN
PATHWAYS IN CANCER	362	-1.4579334	0.15635398	DOWN
SEROTONERGIC SYNAPSE	101	-1.4559821	0.15634629	DOWN
OXYTOCIN SIGNALING PATHWAY	136	-1.4458885	0.16175647	DOWN
PRIMARY IMMUNODEFICIENCY	33	-1.444038	0.16154702	DOWN
HISTIDINE METABOLISM	22	-1.4430106	0.16083072	DOWN
CAMP SIGNALING PATHWAY	162	-1.4323806	0.16688174	DOWN
GALACTOSE METABOLISM	28	-1.4302329	0.16694595	DOWN
OTHER GLYCAN DEGRADATION	17	-1.4271425	0.16777167	DOWN
SALMONELLA INFECTION	72	-1.4261873	0.16696166	DOWN
DOPAMINERGIC SYNAPSE	109	-1.4211104	0.16935831	DOWN
OLFACTORY TRANSDUCTION	54	-1.3909026	0.18913583	DOWN
JAK-STAT SIGNALING PATHWAY	122	-1.3904344	0.18797296	DOWN
VASCULAR SMOOTH MUSCLE CONTRACTION	110	-1.3677438	0.20406732	DOWN
NON-SMALL CELL LUNG CANCER	56	-1.3565415	0.21164037	DOWN
OVARIAN STEROIDOGENESIS	44	-1.354448	0.21186776	DOWN
ANTIGEN PROCESSING AND PRESENTATION	65	-1.3527162	0.21159388	DOWN
INSULIN SECRETION	71	-1.3317658	0.2296786	DOWN
CITRATE CYCLE (TCA CYCLE)	29	-1.3257945	0.23294027	DOWN
GNRH SIGNALING PATHWAY	78	-1.321991	0.23454982	DOWN
NOTCH SIGNALING PATHWAY	47	-1.3187945	0.23573256	DOWN
LEGIONELLOSIS	53	-1.3172578	0.23515317	DOWN
EPSTEIN-BARR VIRUS INFECTION	188	-1.3083749	0.24143504	DOWN
GABAERGIC SYNAPSE	70	-1.2981826	0.2486908	DOWN

Supplementary table 4. Significantly Enriched Gene Sets found in liver of rapamycin fed *Terc*$^{+/+}$ as compared to control fed *Terc*$^{+/+}$ male mice. Source: KEGG. Related to Figure 6. FDR: False Discovery Rate, NES: Normalized Enrichment Score. FDR<0.25

Gene Set Name	Genes	NES	FDR q-val	Rapamycin *Terc*$^{+/+}$ status
FANCONI ANEMIA PATHWAY	48	2.345258	0.04886671	UP
SYSTEMIC LUPUS ERYTHEMATOSUS	94	2.0967045	0.1327473	UP
HERPES SIMPLEX INFECTION	170	2.009204	0.1493091	UP
HOMOLOGOUS RECOMBINATION	28	1.9431006	0.16866498	UP
PROTEIN PROCESSING IN ENDOPLASMIC RETICULUM	160	1.9022516	0.17113815	UP
TERPENOID BACKBONE BIOSYNTHESIS	21	1.8885716	0.1530521	UP
BASE EXCISION REPAIR	33	1.8661711	0.1468089	UP
STEROID BIOSYNTHESIS	17	1.8639425	0.13064268	UP
INFLUENZA A	145	1.8273373	0.14540765	UP
MATURITY ONSET DIABETES OF THE YOUNG	15	1.8174168	0.13862576	UP
STARCH AND SUCROSE METABOLISM	47	1.8021909	0.13589475	UP
MISMATCH REPAIR	22	1.7785974	0.14263324	UP
DRUG METABOLISM CYTOCHROME P450	58	1.7416648	0.16053364	UP
RIBOSOME	127	-5.663769	0	DOWN
FOCAL ADHESION	192	-2.9753597	0	DOWN
ECM-RECEPTOR INTERACTION	76	-2.9567683	0	DOWN
RNA TRANSPORT	153	-2.7942865	0.00160971	DOWN
AGE-RAGE SIGNALING PATHWAY IN DIABETIC COMPLICATIONS	99	-2.4854283	0.00931791	DOWN
PI3K-AKT SIGNALING PATHWAY	296	-2.4818432	0.00800339	DOWN
AXON GUIDANCE	119	-2.4005191	0.00967133	DOWN
NEUROACTIVE LIGAND-RECEPTOR INTERACTION	161	-2.2545912	0.02527886	DOWN
OXIDATIVE PHOSPHORYLATION	110	-2.253554	0.02260518	DOWN
BIOSYNTHESIS OF AMINO ACIDS	72	-2.2192342	0.02366613	DOWN
PROTEOGLYCANS IN CANCER	189	-2.1536725	0.03444197	DOWN
ALANINE, ASPARTATE AND GLUTAMATE METABOLISM	32	-2.071477	0.0511647	DOWN
LYSOSOME	118	-2.0665796	0.04924004	DOWN

PROTEIN DIGESTION AND ABSORPTION	75	-2.0597234	0.04714516	DOWN
ARGININE BIOSYNTHESIS	19	-2.0534775	0.04655975	DOWN
PURINE METABOLISM	161	-2.0321035	0.05013266	DOWN
PHOSPHATIDYLINOSITOL SIGNALING SYSTEM	90	-1.9959034	0.05960937	DOWN
MICRORNAS IN CANCER	132	-1.9893808	0.0582199	DOWN
T CELL RECEPTOR SIGNALING PATHWAY	100	-1.9853731	0.05670829	DOWN
GLUTATHIONE METABOLISM	54	-1.973799	0.0576875	DOWN
HUNTINGTON'S DISEASE	171	-1.950863	0.06190695	DOWN
RENAL CELL CARCINOMA	65	-1.9421681	0.06203564	DOWN
GLYCOSAMINOGLYCAN BIOSYNTHESIS CHONDROITIN SULFATE / DERMATAN SULFATE	20	-1.9193283	0.06505505	DOWN
ALZHEIMER'S DISEASE	153	-1.9027333	0.06914794	DOWN
INOSITOL PHOSPHATE METABOLISM	67	-1.877219	0.07719376	DOWN
PATHWAYS IN CANCER	364	-1.8640112	0.07998864	DOWN
RAS SIGNALING PATHWAY	199	-1.8365883	0.09093642	DOWN
HIPPO SIGNALING PATHWAY	139	-1.8318524	0.0900906	DOWN
B CELL RECEPTOR SIGNALING PATHWAY	71	-1.8173939	0.09455697	DOWN
CHRONIC MYELOID LEUKEMIA	73	-1.8150048	0.09267087	DOWN
WNT SIGNALING PATHWAY	128	-1.8140697	0.09003308	DOWN
FC GAMMA R-MEDIATED PHAGOCYTOSIS	84	-1.7977295	0.09541893	DOWN
REGULATION OF ACTIN CYTOSKELETON	195	-1.78462	0.09838633	DOWN
VASCULAR SMOOTH MUSCLE CONTRACTION	110	-1.7835702	0.09642882	DOWN
SMALL CELL LUNG CANCER	81	-1.7787828	0.09588088	DOWN
HEMATOPOIETIC CELL LINEAGE	75	-1.7787211	0.09325552	DOWN
TGF-BETA SIGNALING PATHWAY	79	-1.761478	0.09948144	DOWN
ARGININE AND PROLINE METABOLISM	43	-1.7508593	0.10270624	DOWN
RAP1 SIGNALING PATHWAY	193	-1.7414085	0.10539897	DOWN
PARKINSON'S DISEASE	121	-1.7103263	0.11987763	DOWN
TAURINE AND HYPOTAURINE METABOLISM	10	-1.6865342	0.13273595	DOWN
HYPERTROPHIC CARDIOMYOPATHY (HCM)	73	-1.6399595	0.16091892	DOWN
PENTOSE PHOSPHATE PATHWAY	27	-1.6362396	0.1596185	DOWN

CARDIAC MUSCLE CONTRACTION	62	-1.6342821	0.15732892	DOWN
AMOEBIASIS	96	-1.6329796	0.15474078	DOWN
GAP JUNCTION	76	-1.6111139	0.16799	DOWN
CGMP-PKG SIGNALING PATHWAY	153	-1.5949814	0.17849289	DOWN
CELL ADHESION MOLECULES (CAMS)	128	-1.5834248	0.18458924	DOWN
ARACHIDONIC ACID METABOLISM	73	-1.5826377	0.18144444	DOWN
ENDOCYTOSIS	252	-1.5714114	0.1875434	DOWN
PPAR SIGNALING PATHWAY	75	-1.557327	0.19557764	DOWN
CELL CYCLE	121	-1.55725	0.19194493	DOWN
NOTCH SIGNALING PATHWAY	47	-1.5417403	0.20313649	DOWN
COMPLEMENT AND COAGULATION CASCADES	74	-1.5386951	0.20214	DOWN
ALPHA-LINOLENIC ACID METABOLISM	18	-1.534676	0.20245689	DOWN
RIBOSOME BIOGENESIS IN EUKARYOTES	74	-1.5279396	0.20548344	DOWN
MAPK SIGNALING PATHWAY	232	-1.5248302	0.20472483	DOWN
BETA-ALANINE METABOLISM	27	-1.5120596	0.21308659	DOWN
GLIOMA	62	-1.5041845	0.21664242	DOWN
CENTRAL CARBON METABOLISM IN CANCER	64	-1.4983567	0.21797259	DOWN
SPHINGOLIPID SIGNALING PATHWAY	117	-1.4886078	0.22282079	DOWN
HEDGEHOG SIGNALING PATHWAY	38	-1.4699227	0.23718841	DOWN
NON-SMALL CELL LUNG CANCER	55	-1.4637398	0.0851927	DOWN
