# Supplementary material for: The mTOR pathway is necessary for survival of mice with short telomeres
Source: Nat Commun. 2020 Mar 3;11:1168. doi: 10.1038/s41467-020-14962-1 (PMC7054554; doi:10.1038/s41467-020-14962-1)
Supplement: Supplementary file 3 — Reporting Summary [file 41467_2020_14962_MOESM3_ESM.pdf]

nature research

Corresponding author(s): María Antonia Blasco Marhuenda
Last updated by author(s): Jan 3, 2020

Reporting Summary

Nature Research wishes to improve the reproducibility of the work that we publish. This form provides structure for consistency and transparency in reporting. For further information on Nature Research policies, see Authors & Referees and the Editorial Policy Checklist .

Statistics

For all statistical analyses, confirm that the following items are present in the figure legend, table legend, main text, or Methods section.

n/a	Confirmed	
☐	☒	The exact sample size (*n*) for each experimental group/condition, given as a discrete number and unit of measurement
☐	☒	A statement on whether measurements were taken from distinct samples or whether the same sample was measured repeatedly
☐	☒	The statistical test(s) used AND whether they are one- or two-sided<br>*Only common tests should be described solely by name; describe more complex techniques in the Methods section.*
☒	☐	A description of all covariates tested
☐	☒	A description of any assumptions or corrections, such as tests of normality and adjustment for multiple comparisons
☐	☒	A full description of the statistical parameters including central tendency (e.g. means) or other basic estimates (e.g. regression coefficient) AND variation (e.g. standard deviation) or associated estimates of uncertainty (e.g. confidence intervals)
☐	☒	For null hypothesis testing, the test statistic (e.g. *F*, *t*, *r*) with confidence intervals, effect sizes, degrees of freedom and *P* value noted<br>*Give P values as exact values whenever suitable.*
☒	☐	For Bayesian analysis, information on the choice of priors and Markov chain Monte Carlo settings
☒	☐	For hierarchical and complex designs, identification of the appropriate level for tests and full reporting of outcomes
☒	☐	Estimates of effect sizes (e.g. Cohen's *d*, Pearson's *r*), indicating how they were calculated

Our web collection on statistics for biologists contains articles on many of the points above.

Software and code

Policy information about availability of computer code

Data collection	No software was used
Data analysis	Graphpad Prism version 5.0

For manuscripts utilizing custom algorithms or software that are central to the research but not yet described in published literature, software must be made available to editors/reviewers. We strongly encourage code deposition in a community repository (e.g. GitHub). See the Nature Research guidelines for submitting code & software for further information.

Data

Policy information about availability of data

All manuscripts must include a data availability statement. This statement should provide the following information, where applicable:

- Accession codes, unique identifiers, or web links for publicly available datasets
- A list of figures that have associated raw data
- A description of any restrictions on data availability

Source data for figure 1-4, 7 and Supplementary Figure 1-7 are provided with the paper. RNAseq data has been deposited in GEO database (GSE127475) [https://www.ncbi.nlm.nih.gov/geo/query/acc.cgi?acc=GSE127475].

Field-specific reporting

Please select the one below that is the best fit for your research. If you are not sure, read the appropriate sections before making your selection.

☒ Life sciences ☐ Behavioural & social sciences ☐ Ecological, evolutionary & environmental sciences

Life sciences study design

All studies must disclose on these points even when the disclosure is negative.

Sample size	The number of mice used was chosen sufficiently large to reach statistical significance if so. No computational analysis was used under the experimental design. We followed the three Rs guiding principles for more ethical use of animal testing.
Data exclusions	Few animals that suffered from dermatitis had to be sacrificed and excluded from aging/surival curves.
Replication	Aging and survival curve experiments have not been independently replicated due to ethical, time and economic reasons. The sample sizes were sufficiently large to obtain reliable results.
Randomization	The mice were randomly allocated within the groups (control and rapamycin treated).
Blinding	Data analysis was performed blindly.

Reporting for specific materials, systems and methods

We require information from authors about some types of materials, experimental systems and methods used in many studies. Here, indicate whether each material, system or method listed is relevant to your study. If you are not sure if a list item applies to your research, read the appropriate section before selecting a response.

Materials & experimental systems

n/a	Involved in the study	
☐	☒	Antibodies
☒	☐	Eukaryotic cell lines
☒	☐	Palaeontology
☐	☒	Animals and other organisms
☒	☐	Human research participants
☒	☐	Clinical data

Methods

n/a	Involved in the study	
☒	☐	ChIP-seq
☒	☐	Flow cytometry
☒	☐	MRI-based neuroimaging

Antibodies

Antibodies used	mouse monoclonal to phospho-histone H2AX (ser139) (JBW301, Millipore, Cat#05-636), rat monoclonal to p21 (291H/B5), rat monoclonal to p53 (POE316 A/E9), rabbit polyclonal to phospho-histone H3 (Abcam, Cat#ab5168), rat monoclonal to p19 ARF (5-C3-1, Santa Cruz Biotechnology, Cat#sc-32748) or rabbit polyclonal to AC3 cleaved-caspase 3 (Asp175) (Cell Signaling Technology, Cat#9661), rabbit polyclonal anti phospho-S6 ribosomal protein (Ser240/244) (Cell Signaling Technology, Cat#2215),anti LC3 (Cell Signaling Technology, Cat#2775), anti-β-actin (Sigma), anti-SMC1 (Bethyl), anti-total OXPHOS (Total OXPHOS Rodent WB Antibody Cocktail, Cell Signaling Technology, Cat#110413).
Validation	Validation of the antibodies are available in company's website or in the CNIO Histopathology Unit repository.

Animals and other organisms

Policy information about studies involving animals; ARRIVE guidelines recommended for reporting animal research

Laboratory animals	Mouse musculus, C57BL/6 background
Wild animals	This study does not involve wild animals
Field-collected samples	This study does not involve samples collected from the field
Ethics oversight	All animal experiments were approved by the Ethical Committee (CEIyBA) (IACUC.040-2014, CBA_20_2014) and performed in accordance with the guidelines stated in the International Guiding Principles for Biomedical Research Involving Animals, developed by the Council for International Organizations of Medical Sciences (CIOMS).

Note that full information on the approval of the study protocol must also be provided in the manuscript.
